# Association between past oral contraceptive use and the prevalence of hypertension in postmenopausal women: the fifth (2010–2012) Korea National Health and Nutrition Examination Survey (KNHANES V)

**DOI:** 10.1186/s12889-021-12410-3

**Published:** 2022-01-06

**Authors:** JungJu Lee, Hyunsuk Jeong, Joo Hee Yoon, Hyeon Woo Yim

**Affiliations:** 1 Department of Medicine, College of Medicine, Catholic University of Korea, Seoul, Korea; 2 Department of Preventive Medicine, College of Medicine, The Catholic University of Korea, 222 Banpodero, Seochogu, Seoul, 06591 Korea; 3 Department of Obstetrics and Gynecology, College of Medicine, St. Vincent’s Hospital, The Catholic University of Korea, Suwon, Republic of Korea

**Keywords:** Oral contraceptive, Hypertension, Postmenopausal women

## Abstract

**Background:**

There is little evidence as to whether the use of oral contraceptives(OC) during the fertile years affects the development of postmenopausal hypertension. This study aimed to evaluate the association between past use of OC and development of hypertension in postmenopausal women.

**Methods:**

This was a cross-sectional study conducted using data from the Fifth Korea National Health and Nutrition Examination Survey of postmenopausal women. Subjects were classified into three groups based on past OC use duration: nonusers, short-term users(0–30 months), and long-term users(≥ 30 months). We evaluated the development of hypertension in women after menopause. A multivariable logistic regression model was used to identify the association between the use of OC during the fertile years and the prevalence of hypertension after menopause following adjustment for potential confounding factors.

**Results:**

Of the 3,386 postmenopausal women, 2,713 were nonusers of OC, 489 were short-term users, and 184 were long-term users. Women who had used OC for 30 months or more had a significantly greater prevalence of hypertension after menopause than those who had never taken OC. The association between taking OC for 30 months or more during the fertile years and the prevalence of hypertension after menopause was significant following adjustment for potential confounding factors (adjusted OR:1.75; 95%CI:1.12–2.74).

**Conclusion:**

This study identified an association between past OC use and an increased prevalence of hypertension in postmenopausal women. Our results suggest that long-term use of OC during the fertile years can be an important risk factor for subsequent hypertension after menopause.

**Supplementary Information:**

The online version contains supplementary material available at 10.1186/s12889-021-12410-3.

## Introduction

Oral contraceptives (OCs) were introduced in the 1960s as the most widely accepted contraception method in the world. Recent studies have shown that OC use is associated with an increased risk of cardiovascular diseases such as myocardial infarction [1], stroke [1], venous thrombosis [2], and hypertension [3]. However, these studies have mainly considered current use of OC in young women of reproductive age.

Hypertension places the largest health burden on people over 50 years old worldwide of any disease [4] and, in particular, the prevalence of hypertension in women is increasing faster than that in men [5]. Hypertension is a major cause of women's cardiovascular disease morbidity and mortality, which is now a major public health concern [5].

Endogenous estrogen is thought to protect women from vascular disease and atherosclerosis [6], while exogenous estrogen is associated with an increased risk of stroke [7] and hypertension [8]. The risk of hypertension increases rapidly during menopause, which is a time when women undergo hormonal changes. An epidemiological study has shown that the prevalence of hypertension in men is higher than in women during the young adulthood period, while that in women increases significantly after menopause [9]. Therefore, it is necessary to identify factors influencing the development of hypertension in women after menopause.

A cross-sectional study conducted in Australia reported that past use of OC in postmenopausal women was not associated with postmenopausal development of hypertension [10]. However, in this study, since there was no distinction between whether hypertension occurred before or after menopause and because it is not known whether hypertension or OC use came first, it is difficult to determine from this prior study whether using OC during the fertile years increases the risk of postmenopausal hypertension.

This study used data from the Korea National Health and Nutrition Examination Survey (KNHANES) conducted from 2010 to 2012 to analyze the association between past use of OC and the occurrence of postmenopausal hypertension. A previous study examined the relationship between OCs use and blood pressure and the prevalence of hypertension in aged 35–55 years’ Korean women [11]. Since the questionnaire about OCs use does not ask the current use of OC, but the past experience and total duration of the OCs use in the KNHANES, the possibility of reverse causation cannot be ruled out in explaining the association between OC use and the development of hypertension. Although KNHANES is a cross-sectional survey, we tried to clarify the temporal relationship between OC use and the onset of hypertension by excluding the participants diagnosed with hypertension prior to menopause in the current study. Therefore, we examined to the effect of past OC use and development of hypertension after menopause. In addition, we aimed to investigate whether OC use during childbearing years had a long-term effect on the development of postmenopausal hypertension and whether the risk of hypertension development was different depending on the duration of OC use.

## Methods

### Study population

The KNHANES uses a complex, multistage (three-stage) probability sample design. The sample represents the total noninstitutionalized civilian population of Korea. In this context, the primary sample units (PSUs) for the KNHANES are selected from a sampling frame of all census blocks or resident registration addresses. Each PSU consists of approximately 50 to 60 households. Following the selection of PSUs, all dwelling units in the PSU are listed and 20 households are selected through the field survey for household screening. The final stage of selection occurs in the household, where all members aged one year or older are selected to participate. Approximately 10,000 persons are sampled in total in all 192 PSUs per year.

We analyzed KNHANES V (2010–2012) data for the current study. Of the 25,534 participants, 5,188 were postmenopausal women. Participants with a history of early menopause (onset < 40 years), postsurgical or medical amenorrhea (e.g., due to hysterectomy or the use of intrauterine devices), or hormone replacement therapy were excluded. Participants with incomplete key data—including those concerning the duration of OC use, age at the time of hypertension diagnosis, age at menopause onset, and measured blood pressure values—were also excluded. To assess the association between OC use and the incidence of hypertension after menopause, participants diagnosed with hypertension prior to menopause were excluded.

**Fig. 1:**
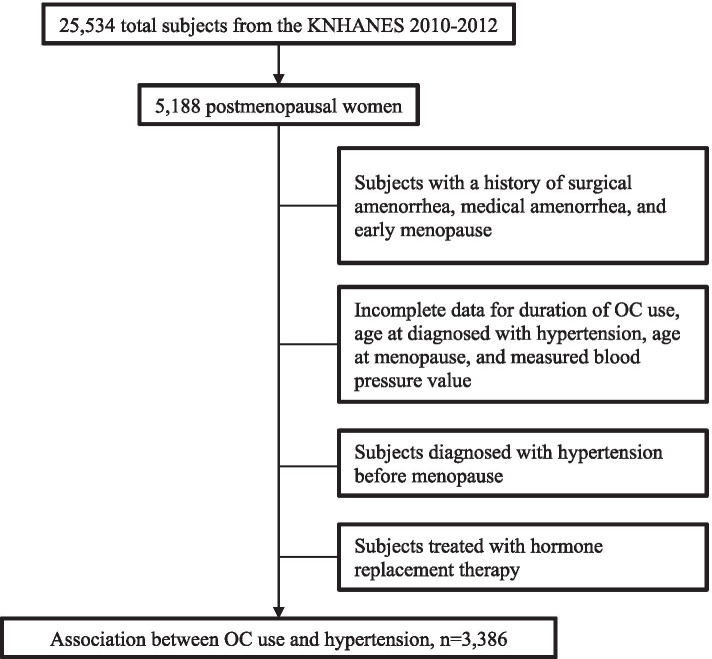
Flowchart of the study participant selection process

A total of 3,386 women after menopause were included in the current analysis. The flowchart showing the subject selection process is presented in Fig. 1.

## Measurements

### Definition of the dependent variable

Hypertension was defined for this study based on the seventh report of the Joint National Committee (JNC7) as a systolic blood pressure (BP) (SBP) greater than 140 mmHg, diastolic blood pressure (DBP) greater than 90 mmHg, and/or antihypertensive medicine being taken [12]. BP was measured three times by the cuff of the right arm attached to the Mercury sphygmomanometer (Baumanometer; W. A. Baum, Copiague, NY, USA). The final BP was calculated as the average of the second and third recorded values.

### Definition of independent variables

The duration of OC use, an independent variable, was investigated through a self-reported health questionnaire. If participants answered “yes” to the question "have you ever taken oral contraceptives for at least one month?”, they were asked to enter the total duration of OC use in years and months. In this study, participants were classified into three groups according to their past use pattern of OC as follows: never, less than 30 months, and 30 months or more.

### Potential confounding factors

For the confounding variables selection process, we listed the variables with statistically significant association with OC use and known causal association with hypertension through the existing literature review. Among them, we selected minimal sufficient adjustment sets for estimating the association between OC use and prevalence of hypertension after menopause using the Directed acyclic graphs (DAG). Based on the DAG, we adjusted age, BMI, education level, household income, smoking status, alcohol consumption, physical activity, DM, dyslipidemia, age at menopause, and number of pregnancies in the final multivariable model (Supplementary Fig. 1).

Potential confounding factors, including age, body mass index (BMI), current smoking status, high-risk drinking status, physical activity, diabetes mellitus (DM), hyperlipidemia, age at menopause onset, and number of pregnancies were derived from demographic and personal medical data. Education level was categorized into three groups: middle school or below, high school, or university graduation. A current smoker was defined as an individual who has smoked more than five packs of cigarettes (100 cigarettes) in her lifetime and who now smokes. Conversely, people with five packs or less of a lifetime of smoking were considered nonsmokers. Heavy drinking was defined as having more than five drinks at a time on average and drinking more than twice per week. According to the recommendations of the American College of Sports Medicine and the American Heart Association, adequate physical activity was defined as performing 20 min of vigorous-intensity physical activity three or more days per week or 30 min of moderate-intensity physical activity five or more days per week [13].

### Statistical analysis

All continuous data were expressed as mean ± standard error and all categorical data were expressed in numbers and percentages. The sociodemographic characteristics between the three groups were compared using analysis of variance (ANOVA) for continuous variables or the Rao–Scott chi-square test for categorical variables. To analyze the association between the use of OC during the fertile years and the prevalence of hypertension after menopause, odds ratios (ORs) for hypertension prevalence according to OC usage were estimated in the groups taking OC for less than 30 months and 30 months or more, respectively, using univariable logistic regression and multivariable logistic regression. Model 1 presents crude ORs, while model 2 presents adjusted ORs after adjusting for age and BMI. Finally, model 3 was adjusted according to age, BMI, education level, household income, physical activity, excessive drinking, smoking status, DM, hyperlipidemia, age at menopause onset, and number of pregnancies. In addition, the association between the duration of OC use and the prevalence of hypertension was analyzed using univariable and multivariable logistic regression analyses.

Survey variables that incorporated weighting, clustering, or stratification were used in the statistical analysis to produce national estimates for the OC use and prevalence of hypertension after menopause based on the survey design for KNHANES. Unweighted numbers were reported in Table 1 to provide descriptive measures of the study participants. In order not to confuse the reader, we added this information to the statistical analysis section. We checked to see if any of the variables included have a high correlation (about 0.7 or higher) with any other variable. As we can see, upon review of this correlation matrix, there does not appear to be any variables with a particularly high correlation. All statistical analyses were analyzed by two-tailed tests and were considered statistically significant for P < 0.05. Weighted population samples were used in consideration of sampling methods and response rates, and all statistical analysis was conducted using SAS version 9.4 (SAS Institute, Cary, NC, USA).

## Results

### General characteristics of the participants

The study participants' general characteristics are given in Table 1. Overall, 2,713 postmenopausal women had no past history of OC use, while 673 had experience using OC in the past, including 184 of whom had used OC for 30 months or more. The average period of OC use was 10.1 months for those who had used OC less than 30 months but 65.4 months for those who had used OC for 30 months or more. The average BMI was 24.1 kg/m^2^ in the group that had never used OC, 24.3 kg/m^2^ in the group of OC use for less than 30 months, and 24.9 kg/m^2^ in the group of OC use for 30 months or more, with significant differences existing between these three groups in this regard. Considering lifestyle behaviors, the frequency of heavy drinking was 1.0% in the OC nonuse group, 0.8% in the group of OC use for less than 30 months, and 4.2% in the group of OC use for 30 months or more (Table 1).

**Table 1:** General characteristics by duration of OC use (*N* = 3,386)

Characteristics	Duration of OC use			*P*-value
	Never user (*n* = 2,713)	< 30 months (*n* = 489)	≥ 30 months (*n* = 184)	
Demographic factors				
Age (years)	64.1 ± 0.3	64.4 ± 0.5	65.0 ± 0.8	0.544
BMI (kg/m^2^)	24.1 ± 0.1	24.3 ± 0.2	24.9 ± 0.3	0.013
Quartile of monthly household income (*n*, %)				0.629
Upper	651 (22.1)	111 (21.2)	43 (23.8)	
Middle-high	671 (25.9)	118 (23.4)	44 (20.7)	
Middle-low	673 (26.1)	134 (29.0)	46 (24.0)	
Low	685 (25.9)	120 (26.4)	50 (31.4)	
Education level				0.198
≤ Middle school	2097 (78.3)	405 (79.6)	161 (87.8)	
High school	456 (16.0)	67 (16.0)	19 (8.9)	
≥ University	160 (5.7)	17 (4.4)	4 (3.3)	
Lifestyle and behavioral factors				
Current smoker	98 (4.2)	16 (5.6)	6 (4.6)	0.583
Heavy drinker	25 (1.0)	4 (0.8)	3 (4.2)	0.018
Adequate physical activity	403 (14.8)	69 (12.8)	25 (12.5)	0.518
DM (*n*, %)	346 (14.3)	76 (15.0)	32 (19.8)	0.297
Dyslipidemia (*n*, %)				
Age at menopause (years)	49.7 ± 0.1	49.9 ± 0.2	49.4 ± 0.4	0.548
Number of pregnancies	4.9 ± 0.1	5.4 ± 0.1	5.9 ± 0.3	0.055

All data are expressed as mean ± standard error or as number (%)

All *P*-values were calculated using ANOVA or the Rao–Scott chi-square test

*OC* oral contraceptive, *BMI* body mass index, *DM* diabetes mellitus

### Association between past use of OCs and postmenopausal hypertension

**Fig. 2:**
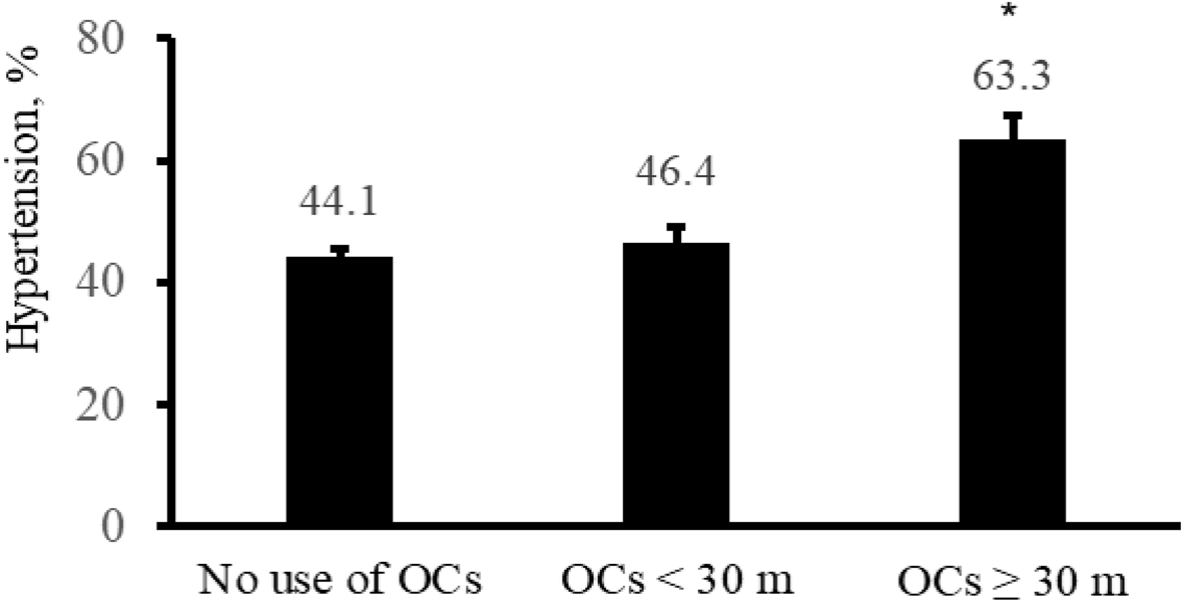
Prevalence of hypertension after menopause according to the period of OC use ^*^P < 0.01

After menopause, the prevalence rate of hypertension among women was 44.1% in the nonuse group, 46.4% in the OC use for less than 30 months group, and 63.3% in the OC use for 30 months or more group (Fig. 2). The ORs for postmenopausal hypertension prevalence in women are shown in Table 2 according to past OC use. In the unadjusted model, the OR for hypertension in the group using OC for 30 months or more was 2.18 [model 1: OR: 2.18, 95% confidence interval (CI) 1.53–3.12]. In model 2, where age and BMI were adjusted, the OR for hypertension in the group using OC for 30 months or more as compared with the nonuser group was 2.11 (model 2: OR: 2.11, 95% CI: 1.31–3.42). The association of hypertension in women after menopause with long-term OC use in the past was significant (model 3: OR: 1.75, 95% CI: 1.12–2.74); however, the OR for hypertension in the group using OC for less than 30 months as compared with the nonuser group was not statistically significant (model 3: OR: 1.05, 95% CI: 0.80–1.37) after controlling for age, BMI, and potential confounding factors (i.e., physical activity, heavy drinking, smoking status, DM, hyperlipidemia, age at menopause onset, and number of pregnancies) (Table 2).

**Table 2:** ORs for the prevalence of hypertension in postmenopausal women according to past use of OC

Duration of OC use					
	No. (*n* = 2713)	< 30 months (*n* = 489)		≥ 30 months (*n* = 184)	
		OR (95%CI)	*P* value	OR (95%CI)	*P* value
Model 1	1 (ref)	1.10 (0.86–1.40)	0.457	2.18 (1.53–3.12)	< 0.001
Model 2	1 (ref)	1.04 (0.81–1.34)	0.746	2.11 (1.31–3.42)	0.002
Model 3	1 (ref)	1.05 (0.80–1.37)	0.166	1.75 (1.12–2.74)	0.021

Model 1: unadjusted; model 2: adjusted for age and BMI; model 3: adjusted for age, BMI, education level, household income, smoking status, alcohol consumption, physical activity, current medical status (DM and dyslipidemia), and reproductive factors (age at menopause and number of pregnancies)

### Association between the duration of OC use and the prevalence of hypertension after menopause onset

The association between the duration of OC use and the prevalence of hypertension is shown in Table 3. In the unadjusted model, the duration of OC use was positively associated with the risk of hypertension after menopause. This association remained significant even after adjusting for age, BMI, lifestyle behaviors, current medical status, and reproductive factors. Moreover, the risk of hypertension in postmenopausal women showed a significant increase of 0.6% per one month of OC use (model 3: OR: 1.006; 95% CI: 1.001–1.011; *P* = 0.015) (Table 3).

**Table 3:** Association between duration of OC use and prevalence of hypertension (*N* = 3,386)

	Duration of OC use	*P*-value
Model 1	1.008 (1.001–1.015)	0.029
Model 2	1.008 (1.002–1.013)	0.011
Model 3	1.006 (1.001–1.011)	0.015

Model 1: unadjusted; model 2: adjusted for age and BMI; model 3: adjusted for age, BMI, education level, household income, smoking status, alcohol consumption, physical activity, current medical status (DM and dyslipidemia), and reproductive factors (age at menopause and number of pregnancies)

## Discussion

This nationally representative, population-based study demonstrated that the prevalence of hypertension after menopause was significantly higher in women who used OC for 30 months or more during their fertile years than in women who did not use OC.

The Nurses’ Health Study, a prospective cohort study, found that current OC users had a significantly increased risk of hypertension relative to those who had never used OC [14]. In addition, several prospective studies have repeatedly reported an increase in blood pressure from the current use of OC [15, 16]. These studies have also consistently demonstrated that these effects decrease rapidly when OC use is stopped [15, 16]. Meanwhile, a few studies have focused on the relationship between past OC use and the subsequent risk of hypertension. The Nurse's Health Study reported that past OC use and use periods did not increase women's mortality or risk of cardiovascular disease, stroke, or coronary artery disease [17, 18]. A secondary (Nurse's Health Study II) investigation reported only a slight increase in the risk for hypertension in past OC users [14]. The 45 and Up Study, a cross-sectional study conducted in Australia, reported that past OC use was not associated with postmenopausal development of hypertension [10]. This result is contrary to our findings. One of the reasons why our research has shown different results from this study is as follows. Although our study is a cross-sectional study, it was able to analyze the association between OC use during the fertile years and postmenopausal hypertension development. It was because that, in addition to current hypertension diagnosis, the ages of hypertension and menopause onset were investigated and we defined cases of hypertension after menopause as events and analyzed them. However, the existing study did not distinguish its population by the timing of hypertension development.

According to experimental evidence, the renin–angiotensin system has a role as a mechanism for hypertension triggered by estrogen. When estrogen (ethinyl estradiol) was administered to a rat model for 12 weeks, hypertension occurred and levels of angiotensinogen and angiotensin II increased. Hypertension caused by estrogen responded to treatment with angiotensin-converting enzyme inhibitors. In other words, estrogen increases the synthesis of angiotensin in the liver and the increase in angiotensin II activates RAAS, triggering sodium reabsorption and water retention, resulting in hypertension [19]. However, if one takes OC for a relatively short period of time such as above, the blood pressure will usually drop to its current baseline within weeks when the OC is stopped [15, 16]. In the current study, the incidence of postmenopausal hypertension was significantly higher in long-term users with past OC use for 30 months or more, but had no effect on those who took it for less than 30 months. A previous study reported that there was vulnerable group of individuals whose blood pressure remained high and progressed to chronic hypertension in past OC users, which may have been prematurely triggered by OCs [20]. Endogenous estrogen secreted from women's ovaries in fertile ages contributes to maintaining the basal vasodilatory state. After menopause, the secretion of endogenous estrogen decreases rapidly, which promotes the development of hypertension in postmenopausal women. In women who took OC for a long time during fertile ages, possibly OC had triggered hypertension prematurely, and endogenous estrogen, a protective factor of hypertension, rapidly decreased after menopause, leading to development of chronic hypertension.

Previous studies have shown a risk of developing high blood pressure even with low dose estrogen preparations [14], especially relative to the duration of use [3]. A meta-analysis demonstrated that the use of OCs increases the risk of hypertension every 5 years by 13% [3]. Another meta- analysis reported that OCs use have been associated with long term adverse cardiovascular effects, such as stroke and coronary artery disease [21].

The dose and type of progestin might influence the incidence rates of hypertension but women taking progesterone-only preparations show few increases in BP. Drospirenone, which is a new fourth generation progestin with antimineralocorticoid diuretic effects, seems to reduce BP when combined to estrogen. In a randomized study, 80 women received the combination drospirenone 3 mg plus ethinylestradiol 30, 20 or 10 μg or levonorgestrel 150 μg plus ethinylestradiol 30 μg during six cycles [5]. Systolic and diastolic BP reduced by 1–4 mm Hg with any of drospirenone combinations and increased by 1–2 mm Hg with levonorgestrel combination [5].

There are several limitations in this study. First, the cross-sectional study design did not allow us to draw solid conclusions regarding the causal relationships between OC use and postmenopausal hypertension development. In this study, hypertension after menopause, the primary study outcome, was defined in consideration of the age at hypertension diagnosis and age at menopause onset, while the use of OC, the exposure factor, is generally performed during the fertile years, so the time relationship between exposure and outcome is clear. Second, this study relied on self-reported data, with potential information bias, so data concerning hypertension, menopause, and the duration of OC use may be under- or over-reported. Especially, hypertension is an asymptomatic disease that can mislead the assumption of the onset of disease. Self-reported medical history of blood pressure is probably not accurate even though blood pressure checked biannually for all of the people over 40 years of age through a national health examination in Korea. However, information bias would have triggered nondifferential misclassification between the OC use and nonuse groups. Both the OC-exposed and -nonexposed groups were designed without knowing the research hypothesis at the time of investigation, so equal misclassification in measurements of both the exposure and outcome variables might have occurred in the current study. Third, even though the potential confounding factors have been adjusted, there may be residual confounding due to other unexamined factors known to affect the occurrence of hypertension, including type and dose of OC. The women included in this study were those exposed to early-generation OC, and the results of this study are likely to reflect an association with higher dose formulations. Later-generation OC contains lower amounts of estrogen and progestin as well as new combinations of hormones [22]. Although recent studies using low-capacity contraceptives have reported reduced effects on the risk of cardiovascular disease, there remains a significant risk [23]. Fourth, in some cases, oral contraceptives used for purposes other than contraception, such as dysfunctional uterine bleeding, dysmenorrhea, menorrhagia, endometriosis, or functional ovarian cyst. Patients with endometriosis have a higher risk of hypertension. It may be a higher risk group with other underlying diseases. Other options of contraception such as Mirena and emplaning can increase blood pressure. However, these data were not collected in KNHANES, we are unable to ascertain them. Fifth, hypertension is linked to female sexual dysfunction. However, the group who did not take contraceptive agents because of sexual dysfunction could not be identified in this study. Sixth, because the age of OC uses was not collected in KNHANES survey, we are unable to analysis whether the age of OC uses affect occurrence of hypertension after menopause. Finally, we did not include some dietary factors—intake of sodium, potassium, and fruits and vegetables—which have been kwon as risk factors for blood pressure as potential confounders in the final model.

Also, the current study had several strengths. First, our investigation provided evidence of the close relationship between OC use in the fertile years and postmenopausal hypertension development. Second, this was a population-based analysis using a well-studied, nationally representative sample data that enhances the statistical reliability of the results and the generalizability of the data.

Women who want to take OC during their fertile years should be warned of an increased risk of hypertension after menopause and each should carefully examine the risks and benefits of OC use in consultation with their physician before deciding whether to take them.

## Conclusion

In conclusion, this study identified an association between the use of past OCs and an increased prevalence of hypertension in postmenopausal women. The results suggest that long-term use of OC in the fertile years may be an important risk factor for subsequent hypertension development. Further research is needed to determine the long-term effects of OC on the risk of hypertension. Since past OC use may be one of the causes of postmenopausal hypertension, providers should consider a patient’s blood pressure control and overall cardiovascular health when discussing the risks and benefits of contraceptive methods as compared to the risks of an unintended pregnancy in the clinical setting.

## Supplementary Information

**Additional file 1.** Directed acyclic graph (DAG) in our study.

## Data Availability

The original study data are publicly available for free on the KNHANES website (https://knhanes.kdca.go.kr/knhanes/sub03/sub03_02_05.do).

## Abbreviations

KNHANES: Korea National Health and Nutrition Examination Survey
OC: Oral contraceptives
PSU: Primary sample unit
IRB: Institutional Review Board
JNC7: The seventh report of the Joint National Committee
BP: Blood pressure
SBP: Systolic blood pressure
DBP: Diastolic blood pressure
BMI: Body mass index
DM: Diabetes mellitus
ANOVA: Analysis of variance
OR: Odds ratio
